# Sleep Disorders in Patients with Tics: Towards Personalized Care for Tourette Syndrome

**DOI:** 10.3390/jpm16060309

**Published:** 2026-06-06

**Authors:** Kashish K. Qureshi, Andrea E. Cavanna

**Affiliations:** 1 Department of Neuropsychiatry, Birmingham and Solihull Menatal Health NHS Foundation Trust and School of Medical Sciences, College of Medicine and Health, University of Birmingham, Birmingham B15 2FG, UK; kashishqureshi2003@gmail.com; 2 School of Health and Life Sciences, Aston Institute of Health and Neurodevelopment, Aston University, Birmingham B4 7ET, UK; 3 Sobell Department of Motor Neuroscience and Movement Disorders, Institute of Neurology and University College London, London WC1E 6BT, UK; 4 School of Medicine and Surgery, University of Milano-Bicocca, 20125 Milan, Italy

**Keywords:** sleep, sleep disorders, tic disorders, tics, Tourette syndrome

## Abstract

**Background/Objectives**: Tourette syndrome and other chronic tic disorders are neurodevelopmental conditions characterized by intermittent motor/phonic tics and frequent behavioral comorbidity. Poor sleep quality is often reported by patients with tic disorders; however, little is known about the prevalence and clinical correlates of disruption in sleep physiology. **Methods**: We conducted a systematic literature review of clinical studies evaluating sleep using at least one validated sleep outcome (questionnaire, polysomnography, or coded clinical diagnosis). **Results**: Despite high heterogeneity in age ranges, diagnostic formulations, outcome measures, and confounder handling, converging evidence across designs indicated a significantly higher prevalence of sleep disturbance in patients with Tourette syndrome and other chronic tic disorders compared to controls. Specifically, registries showed significantly greater insomnia rates (aOR 6–7); case–control studies revealed a 9-fold increase in night-waking, bedtime resistance, parasomnias, and daytime drowsiness; polysomnography studies demonstrated sleep fragmentation, with decreased efficiency, longer latency, and more awakenings. **Conclusions**: Sleep disorders are relatively common in patients with Tourette syndrome and other chronic tic disorders, with clinical implications for both arousal instability and sleep initiation/maintenance issues. Further research is needed to better understand the complex interplay between altered sleep patterns and tic expression, as well as the impact of behavioral comorbidities. Our findings highlight a need for personalized treatment interventions focusing on sleep problems in the context of tic disorders.

## 1. Introduction

Tourette syndrome (TS) and other chronic tic disorders (TDs) are neurodevelopmental conditions characterized by tics, defined as sudden, rapid, recurrent, and non-rhythmic movements or vocalizations [1]. Specifically, TS is defined by the occurrence of multiple motor tics plus at least one vocal tic, with onset before the age of 18 [2]. The other TDs are considered to be truncated forms of TS within the broader concept of the “TS spectrum” [3,4]. Provisional tic disorder is characterized by tics that last less than a year, whereas persistent motor or vocal tic disorders are characterized by the chronic presence of either motor or vocal tics (but not both) [2].

TS is currently classified as a neuropsychiatric disorder with a strong genetic component, typically emerging in childhood, peaking in severity in adolescence, and frequently improving into adulthood [5,6,7]. The estimated prevalence of tic disorders in childhood is approximately 0.77%, with a male-to-female ratio of 4:1 [8]. A considerable proportion of patients report complex manifestations—complex motor tics involving multiple muscles, possibly resembling compulsive behaviors [9], and/or complex vocal tics, including coprolalia [10]. In addition to tics, a wide range of psychiatric comorbidities are commonly reported, including tic-related obsessive–compulsive disorder (OCD), attention-deficit/hyperactivity disorder (ADHD), anxiety, affective disorders, and impulse control disorders [11,12,13,14,15,16,17]. These co-occurring conditions significantly contribute to overall functional impairment, decrease health-related quality of life, and complicate clinical management [18,19,20].

Sleep disturbances have increasingly been recognized as a frequent and clinically relevant comorbidity in individuals with TS and other TDs [21,22,23,24,25,26,27]. Early clinical observations already hinted at abnormal sleep-related phenomena in this population, including confusional arousals and nocturnal behavioral disturbances [28]. More recent studies indicate that sleep problems are highly prevalent, although reported estimates vary considerably according to study design, population characteristics, and assessment methods. Reported prevalence rates range from less than 10% to up to 80%, highlighting the heterogeneity of findings and the need for more standardized approaches to evaluation [29].

Sleep plays a fundamental role in brain development and function, supporting processes such as synaptic plasticity, memory consolidation, emotional regulation, and executive functioning [30]. Disruption of normal sleep architecture can therefore have profound neurocognitive and behavioral consequences. In individuals with tic disorders, sleep disturbances may exacerbate tic severity, impair daytime functioning, and worsen associated psychiatric symptoms [31]. Moreover, insufficient or poor-quality sleep has been linked to alterations in neurobiological systems, including dysregulation of the hypothalamic–pituitary–adrenal axis, increased sympathetic activity, and changes in inflammatory markers [26]. These mechanisms may contribute to adverse clinical outcomes, including heightened emotional reactivity and increased risk of mood disorders.

The spectrum of sleep disorders observed in TS and other TDs is broad and encompasses multiple categories defined in the Diagnostic and Statistical Manual of Mental Disorders, 5th Edition, text revision (DSM-5-TR) [2]. Insomnia is among the most commonly reported complaints, often manifesting as difficulty initiating or maintaining sleep [23]. Excessive daytime sleepiness is also frequently described, potentially reflecting fragmented nocturnal sleep or underlying neurophysiological dysregulation. Parasomnias, including sleepwalking, sleep talking, night terrors, and enuresis, appear to occur at elevated rates in this population. Additionally, sleep-related movement disorders, such as periodic limb movements during sleep, and the persistence of tics during sleep have been documented through polysomnographic studies.

The relationship between tic disorders and sleep disturbances is complex and likely multifactorial. Evidence suggests that individuals with tic disorders and comorbid conditions experience more severe and more frequent sleep disturbances than those with tic disorders alone [32]. For example, the presence of ADHD has been linked to increased rates of insomnia and sleep fragmentation, while anxiety and mood disorders may contribute to difficulties with sleep initiation and maintenance [24]. Pharmacological treatments, often used to manage tics and comorbid symptoms, may further influence sleep patterns, either positively or negatively.

Despite the growing recognition of the importance of sleep in patients with tic disorders, significant gaps remain in the literature. Variability in prevalence estimates, limited longitudinal data, and a lack of standardized assessment tools hinder the ability to draw definitive conclusions on the nature and etiology of sleep disturbances in this population. Furthermore, the relative contribution of biological, psychological, and environmental factors remains insufficiently understood. Therefore, we conducted a systematic literature review to determine and quantify the prevalence of sleep disorders among individuals with TS and other TDs. We sought to examine differences in reported prevalence rates across age groups, with particular emphasis on potential distinctions between pediatric and adult populations, as well as on the impact of comorbid psychiatric conditions on the type and frequency of sleep disturbances. We focused on the best available evidence and assessed the methodological quality of included studies to evaluate the reliability and validity of the evidence base and to identify common limitations within the field. In this context, a comprehensive evaluation of sleep disturbances in individuals with TS and other TDs can enhance our understanding of underlying mechanisms, clarify the role of comorbidities, and inform the development of targeted (personalized) interventions. Early identification and management of sleep problems are of particular importance, as improving sleep may have beneficial effects on tic severity, emotional regulation, and the health-related quality of life of the individual patient.

## 2. Materials and Methods

This systematic review aimed to synthesize primary evidence on the prevalence of sleep disturbances among individuals with TS and other TDs of any age and from any clinical setting. We followed the methodology outlined in the current version of the PRISMA (Preferred Reporting Items for Systematic Reviews and Meta-Analyses) guidelines [33] to ensure transparent, rigorous, and reproducible reporting (Supplementary File S1). The present systematic literature review was not registered. Since a separate protocol was not prepared, there were no amendments to either the information provided at registration or in the protocol. To identify studies investigating the prevalence, type, and severity of sleep disturbances in TS/TD populations, the search strategy combined free-text keywords with controlled vocabulary terms. To identify studies investigating the prevalence, type, and severity of sleep disturbances in TS/TD populations, the search strategy combined free-text keywords with controlled vocabulary terms. Ovid Medline, Embase, PsycINFO, PubMed, Web of Science, and CINAHL were the six scientific databases used in the search. In addition to relevant MeSH (PubMed) and Emtree (Embase) terms, the search strategy combined the following keywords using Boolean operators: (“Tourette syndrome” OR “tic disorder”) AND (“sleep disturbance” OR “insomnia” OR “restless sleep” OR “sleep latency” OR “REM latency” OR “parasomnia” OR “sleep duration” OR “polysomnography” OR “actigraphy” OR “sleep questionnaire” OR “sleep quality”). Whenever possible, we used wildcard truncations “tic*” and “sleep*” to expand our search. The search was limited to studies published from 2000 onward to focus on investigations using updated diagnostic criteria for tic disorders. In the DSM-IV-TR (2000), the requirement that tics cause functional impairment was removed from the diagnostic criteria for TS [34]. There were no limitations based on gender, ethnicity, or geographic location. The two authors worked independently to determine whether each study met the eligibility criteria until they reached full agreement. Studies that had unclear diagnostic standards or in which the sample for tic disorder could not be distinguished from larger samples (such as “neurodevelopmental disorders” as a composite category) were excluded. Finally, only in a minority of sleep studies were participants drug-naïve or required to discontinue their medication at the time of enrolment. Therefore, we decided to include in our systematic review any studies with patients who were taking pharmacotherapy. Medication use represents an important confounding factor, as central nervous system stimulants may delay sleep onset and reduce total sleep duration, whereas antidopaminergic agents used to treat tics may alter REM sleep architecture. It might prove challenging to separate the direct effects of TS from those of its treatment in the absence of strict drug status and dosage controls.

The methodological quality of the included studies was systematically evaluated using a structured approach designed to accommodate diverse study designs, including cross-sectional, cohort, and case–control studies. To ensure a comprehensive assessment, this review incorporated five domains adapted from the Cochrane Risk of Bias Tool (RoB 2), with a focus on methodological issues relevant to observational clinical research, including participant selection, group comparability, validity of outcome measurement, and statistical rigor [35]. The first domain (selection bias) evaluated the accuracy and representativeness of participant samples. Studies were assessed based on the use of standardized diagnostic criteria and the transparency of recruitment sources (e.g., clinics, schools, or registries). Studies relying on poorly defined or non-representative clinical samples without clear diagnostic procedures were considered at high risk of bias. The second domain (measurement validity) focused on the reliability and appropriateness of sleep assessment methods. Studies employing validated tools—such as actigraphy, polysomnography (PSG), or the Children’s Sleep Habits Questionnaire (CSHQ)—were rated more favorably, particularly when combining subjective and objective measures. In contrast, studies relying exclusively on non-validated, parent-reported questionnaires without blinding were judged to have a higher risk of bias. The third domain (control for confounding variables) included evaluation of whether studies accounted for key demographic and clinical factors, such as age, sex, and common comorbidities, as well as medication use. Studies that implemented strategies such as matching, stratification, or multivariable regression to control for these variables were considered methodologically stronger, while those that failed to address confounding factors were more likely to produce biased results. The fourth domain (statistical integrity) assessed the appropriateness and transparency of analytical methods. This included the use of suitable statistical tests, reporting of effect sizes and confidence intervals, and justification of sample size or statistical power. Studies demonstrating clear and robust statistical reporting were rated more favorably, whereas insufficient methodological detail or incomplete reporting limited interpretability and increased the risk of bias. The fifth domain (reporting and generalizability) evaluated the clarity, completeness, and applicability of study findings. Considerations included sample size adequacy, attrition rates, follow-up completeness, and the extent to which results could be generalized to broader clinical or community populations. Studies with small, single-center samples or substantial attrition were rated as having lower external validity. Each original study was assigned an overall risk of bias rating (low, unclear, or high) based on combined domain assessments. Studies incorporating objective sleep measures and appropriate control of confounders generally demonstrated greater methodological rigor. In contrast, those relying primarily on subjective reports without adequate adjustment for confounding variables exhibited higher risk of bias, despite contributing valuable insights. This structured evaluation enhanced transparency and supported interpretation of the overall strength and reliability of the evidence base.

## 3. Results

**Figure 1:**
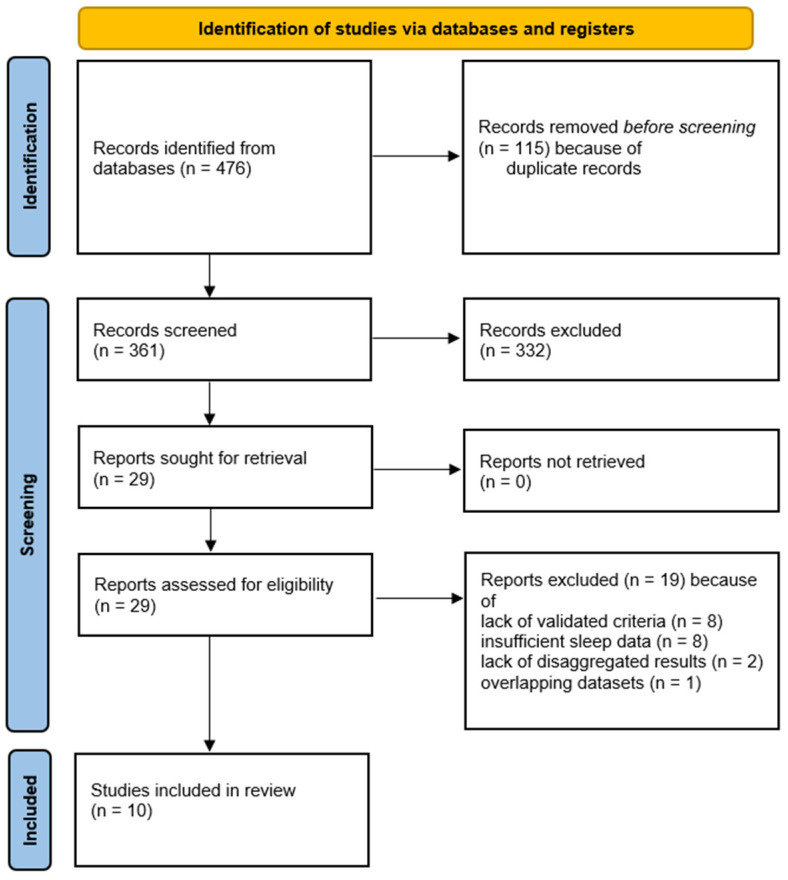
PRISMA 2020 flow diagram showing the study selection process.

The PRISMA flow diagram showing the study selection process of our systematic literature review is presented in Figure 1.

According to RoB 2 ratings, the overall methodological quality of the ten included studies showed a gradient of improvement from 2001 to 2025 (Table 1).

Eight out of the ten reviewed studies were cross-sectional observational studies [36,37,38,39,40,41,42,44]. Of these, three studies incorporated PSG and actigraphy as objective measurements [36,37,38] (Table 2), whereas five reported subjective measures only [39,40,41,42,44] (Table 3). The remaining two were registry-based studies: a population registry study conducted in Sweden [43] and a countrywide cohort with time-to-event analysis carried out in Taiwan [45] (Table 4).

All ten studies reported a high prevalence of sleep disturbances, with estimated rates ranging from 40% to 65%. Prevalence variability was mainly attributed to variations in definitional thresholds, population characteristics, and assessment instruments.

Studies conducted in laboratories (Table 2), namely Cohrs et al. [36], Kostanecka-Endress et al. [37], and Kirov et al. [38], have advantages in terms of impartiality and precision. The use of PSG with synchronized video recording enabled accurate sleep staging, differentiation between tic and non-tic movements, and detection of micro-arousals that may not be captured through subjective reporting. The use of age- and sex-matched healthy controls in these designs improved internal validity by assuring that group differences are due to TS rather than demographic confounds. However, the statistical power and generalizability of laboratory-based PSG investigations was limited by their relatively small sample sizes, which were up to 25 participants per group. Since participation in intense overnight investigations is typically restricted to highly motivated families, these small-scale designs may also be more vulnerable to sample selection bias, which could exclude those with more severe behavioral dysregulation. Moreover, laboratory settings may induce the well-known ‘first-night effect,’ in which sleep architecture is altered because of unfamiliar surroundings. Kostanecka-Endress et al. addressed this limitation by excluding first-night recordings from the analysis [37]. Importantly, the cross-sectional nature of these investigations makes it difficult to determine if the observed changes in sleep are a cause, effect, or co-manifestation of TS and other TDs.

Ecological validity was provided by clinic-based observational studies, namely those by Ghosh et al. [39], Modafferi et al. [40], Sambrani et al. [41], Ricketts et al. [42], and Mi et al. [44] (Table 3). By documenting the real-life experiences of patients and their families, these studies revealed the frequency and type of sleep issues, as well as their practical effects on tic severity, mood, and daytime performance. However, retrospective reports from patients or caregivers—subjective assessments that may not have corresponded to objective sleep parameters and may have been susceptible to recall and social desirability bias. In the study by Ricketts et al., mechanistic interpretation was limited by the single-item measure of “sufficient sleep”, lacking the precision to distinguish between initiation issues, maintenance problems, or early waking [42]. Moreover, in TS populations, the impact of concomitant tic-related OCD, ADHD, anxiety or depression on sleep perception might have exacerbated the well-established disparity between subjective and objective sleep measurements, especially in pediatric patients. A further issue with these clinic-based observational studies was the selection of patients from tertiary referral groups, which might have over-represented subjects with more severe or complex presentations. The generalizability to community samples, where milder or subthreshold cases can be more common, might have been affected by such referral bias.

Externally valid research was best exemplified by large-scale epidemiological investigations, namely the registry-based studies conducted by Isomura et al. [43] and Chung et al. [45] (Table 4). In addition to achieving accurate prevalence estimates and adequate control of confounding variables—sex, birth year, birth country, and physical health problems—the study by Isomura et al. further improved causal inference by using sibling-comparison models, a quasi-experimental approach that accounts for unmeasured familial characteristics including shared genetics and early-life environment [43]. Large follow-up periods were another advantage of registry studies, which allowed for the identification of temporal risk patterns. For example, Isomura et al. analyzed follow-up data for up to 24 years and found that adults with TS or other chronic TDs were more likely to experience insomnia than those with remitting tics [43], whereas Chung et al. analyzed follow-up data for up to 15 years and found that the incidence of sleep disorders was significantly higher in the first year after a TS diagnosis [45]. However, these large registry-based studies necessarily relied on diagnostic codes (based on heterogeneous clinical assessments) and prescription data. Such measurements invariably excluded undiagnosed cases, thereby underestimating true prevalence figures, and might have misclassified illnesses in case of variable coding accuracy. Registry data can also be relatively imprecise: it might be difficult to distinguish between specific forms of sleep disorders (e.g., insomnia versus parasomnias), and it might prove impracticable to gather accurate information on severity, timing, or subjective impact. Furthermore, relying on healthcare utilization records might have increased the likelihood of detection bias: patients with TS might have received more frequent medical attention, resulting in higher recorded rates of comorbidities—including sleep difficulties.

All of the examined studies showed age-related differences in sleep disruption patterns. The most common behavioral issues in younger children were parasomnias, separation anxiety at bedtime, and resistance to bedtime. Adults and teenagers, on the other hand, showed indications of circadian rhythm disturbances, including increased sleep latency, delayed sleep phase syndrome, and higher variability in sleep from workday to weekend. According to the findings of the study by Mi et al., early sleep disruption frequently preceded the start of tics, indicating a prodromal trajectory [44]. Moreover, according to Modafferi et al., sleep problems persisted over a six-month follow-up, suggesting that sleep abnormalities in TS are not temporary, but rather most likely a component of a stable and changing clinical picture [40].

According to population-level data, patients with TS and other TDs had significantly greater odds of sleeplessness, which were further elevated in subgroups with ADHD and in certain strata receiving medication [43]. Similarly, Modafferi et al. found that internalizing symptoms such as worry impacted the severity and chronicity of sleep disturbances [40]. Ricketts et al. provided support for these findings by showing that emotional dysregulation was a stronger predictor of sleep disturbance than tic severity itself [42]. Taken together, these findings suggest that sleep disturbances may be more strongly associated with psychiatric comorbidities than with tic severity alone. However, other studies found that even patients with “pure” forms of TS (without psychiatric comorbidities) have considerable sleep problems, indicating that sleep disturbances are not solely caused by concomitant conditions [36,37]. The results of questionnaire/survey research frequently revealed weak or null relationships between tic severity and sleep outcomes [44], although some PSG metrics demonstrated a correlation with daytime tic severity (e.g., more awakenings and stage shifts in individuals with higher tic severity) [36]. Accordingly, sleep issues and tics might be partially separate symptom domains within the broader neurological framework of TS, even though they regularly co-occur. These findings also highlight the need for clinical assessments and personalized interventions to treat sleep disturbance as a distinct issue rather than a mere collateral effect of tic-related motor activation. Interestingly, although tics can be managed pharmacologically, Isomura et al. reported no discernible improvement in sleep-related symptoms, suggesting that tic suppression does not always resolve sleep problems [43]. Data about anti-tic pharmacotherapy were available only for two studies, Cohrs et al. [36] and Modafferi et al. [40]: in both studies, the most commonly prescribed medications were antidopaminergic agents.

## 4. Discussion

### 4.1. Significance of the Findings

This systematic review analyzed data from ten original studies on the prevalence, types, and clinical factors contributing to sleep disturbances in patients with TS and other TDs. Across the reviewed studies, the prevalence of clinically significant sleep disturbances ranged from 40% to 65%, indicating that sleep abnormalities are a common feature of TS. Registries showed significantly higher insomnia rates compared to the general population (aOR 6–7) [43,45]; case–control studies revealed a 9-fold increase in night-waking, bedtime resistance (the refusal or delay of going to sleep at a scheduled time), parasomnias, and daytime drowsiness [36,37,38,40,42,44]; polysomnography studies demonstrated sleep fragmentation, with decreased efficiency, longer latency, and more awakenings [36,37,38,39]. Sleep disturbances affected multiple domains, including behavioral manifestations (e.g., bedtime resistance and delayed sleep initiation), physiological abnormalities (e.g., REM fragmentation and reduced sleep efficiency), and parasomnia-related symptoms (e.g., hallucinations and sleepwalking). The most commonly reported sleep problems in patients with TS and other TDs are shown in Table 5.

Comorbid mental health disorders—namely tic-related OCD, anxiety, and ADHD—were consistently found to be significant aggravating factors for sleep problems [38,39,40,41,42,43]. Emotional and behavioral dysregulation often outperformed tic severity as indicators of poor sleep, suggesting that these disruptions may be more directly tied to common neurodevelopmental and neurochemical pathways than to direct nocturnal disruption caused by tics. Longitudinal data also showed that the presence of comorbidities was linked to more severe and chronic sleep problems [40].

Age-related differences in sleep disruption patterns showed that adults and adolescents were more likely to have REM-related abnormalities, delayed sleep latency, and circadian rhythm misalignment, while younger children were more likely to present with behavioral and parasomnia-related problems, such as resistance at bedtime and sleepwalking, respectively. According to some data, sleep issues may have preceded the emergence of tics in a subgroup of children, which could indicate that they share prodromal processes or early neurodevelopmental vulnerabilities [44].

Subjective reporting instruments like the CSHQ (a parent-report questionnaire used to assess both behaviorally based and medically based sleep problems in school-aged children) may not always reflect physiological changes indicated by objective evaluations, especially PSG and actigraphy [36,37,38,39]. Shorter REM latency, lower REM density, higher nocturnal arousals, and higher periodic limb movement indices were among these objective findings. Crucially, differences between subjective and objective measurements were often noted. Subjective measures frequently indicated high rates of sleep-onset insomnia, non-restorative sleep, and frequent nighttime awakenings, whereas PSG and actigraphy confirmed delayed sleep onset while showing total sleep time comparable to that of healthy controls. Such differences suggest that clinical evaluation should integrate both approaches to offer a more thorough analysis.

The reviewed literature did not show a regular correlation between the severity of tics and the level of sleep disturbance. Although sleep disturbance in TS and other TDs was found to be consistently higher than in controls across both objective PSG and subjective (questionnaire/registry) assessment modalities, the clinical manifestations of impairment differed, depending on developmental stage, comorbidity pattern, and measuring strategy. These findings support the hypothesis that tic severity and sleep disturbances may reflect partially distinct neurobiological mechanisms. They also suggest that the relationship between tics and sleep disturbances is likely bidirectional rather than solely driven by nocturnal tic activity. While pediatric samples, especially those without comorbid ADHD, did not provide strong evidence for an association between tic severity and sleep problems [38,39], PSG data from adult patients showed lower sleep efficiency and more awakenings correlating with higher tic ratings [36]. This discrepancy can be explained by both developmental features (shifting inhibitory control and comorbidity mix) and methodological factors (laboratory versus carer report; smaller samples undergoing PSG). Increased micro-arousals, persistent (usually simple) nocturnal tics across stages, culminating in lighter NREM, and the frequent discrepancy between subjective complaints and physiological markers could all be mechanistically explained by a hyperarousal/inhibitory-deficit model. A common substrate for higher risk of sleep disturbances that persists in subsets of adults even after tic remission might result from dysregulation in the cortico-striato-thalamo-cortical circuitry and dopaminergic, GABAergic, and serotonergic signaling systems, which are involved in both tic development and sleep–wake regulation. Finally, with some patients displaying sophisticated motor activities during REM sleep, recent studies have also highlighted potential connections between TS and abnormalities similar to RBD [45]. Albeit preliminary, these results might point to pathophysiological pathways shared by tics and parasomnias, which probably deserve further research. These observations have at least two relevant implications for clinical practice. First, sleep quality assessments should purposely combine quick subjective screens with escalation to objective techniques when symptoms persist. Second, possible modifiers should be taken into consideration: both age and comorbidity should dictate intervention thresholds and expected responses.

Large-scale epidemiological studies, population-based surveys, clinic-based observational studies, and laboratory-controlled polysomnographic investigations are all included in the methodologically diverse literature on sleep disruptions in patients with TS and other TDs. One of the field’s most noteworthy advantages is its methodological diversity, which enables the investigation of the issue from several complementary angles, including objective neurophysiological measurement, subjective patient-reported experience, and epidemiological prevalence and risk factors. When analyzing results and combining them into a coherent understanding, it is important to recognize the distinct constraints that each scientific technique brings in addition to its own strengths.

Notably, the reviewed literature often lacks multimodal integration between objective physiological markers and thorough psychological profiles across large, representative samples. Epidemiological findings might not offer significant contributions to the elucidation of pathophysiological mechanisms, whereas the results of mechanistic investigations might not be generalized to the wider population. Furthermore, the vast majority of the studies are cross-sectional, with limitations in terms of causal inference and developmental trajectories—an essential gap given that both tic severity and sleep architecture vary dramatically with age. Lastly, different research groups handle comorbidity differently. While some did not account for these potentially confounding factors, others systematically investigated the role of ADHD and other psychiatric comorbidities [43]. Cross-study comparisons are made more difficult by this variability, which further suggests that observed links between TS and sleep disturbance might not be entirely due to TS, but rather to the influence of highly prevalent comorbidities.

Overall, the reviewed research shows that sleep disruptions in TS are multifaceted, developmentally enduring, and impacted by a complex interaction of behavioral, physiological, psychiatric, and neurobiological variables. Although heterogeneity in assessment methods and study populations limits direct comparison of prevalence estimates, the available evidence provides a multidimensional understanding of how sleep disturbances manifest across the lifespan in individuals with TS. These findings underline the importance of routine and systematic sleep assessments in clinical practice, as well as the potential utility of targeting sleep symptoms as part of a complete TS management strategy.

### 4.2. Possible Treatment Implications

The findings of this systematic review have important clinical implications and support the development of more individualized treatment strategies. First, considering the high frequency of sleep disturbances in all age groups and tic severity levels, routine sleep testing ought to be a basic aspect of TS evaluation. Standardized questionnaires may serve as initial screening tools; however, particularly in complex or treatment-resistant cases, they should be complemented by objective assessments. Second, the need for integrated care strategies that concurrently address tic symptoms and psychiatric comorbidities is highlighted by the significant impact that tic-related OCD, anxiety, and ADHD have on sleep quality.

Both pharmacological and non-pharmacological interventions, as part of individualized treatment strategies, should be considered essential components of TS management [46]. However, to the best of our knowledge, there are no established guidelines for addressing sleep problems in patients with TS and other TDs. Within a personalized care framework, pharmacotherapy options for tics might prove useful for the treatment of associated sleep disturbances. Atypical antipsychotics, characterized by their antidopaminergic properties, are widely used to manage tic symptoms in TS and may also influence associated sleep disturbances. Evidence suggests that these agents may positively influence sleep parameters. For instance, Risperidone has been reported to improve sleep latency and sleep efficiency in a documented case involving a 12-year-old patient [47]. Similarly, Aripiprazole has demonstrated efficacy in reducing tic severity while also contributing to improvements in comorbid psychiatric conditions that can exacerbate sleep-related complaints. Moreover, low-dose Aripiprazole might be effective for treatment of delayed sleep phase syndrome [48]. However, findings are not entirely consistent across different individuals. A prospective, uncontrolled, open-label study involving 44 adults indicated that Aripiprazole may, in some cases, provoke sleep disturbances as an adverse effect [49]. In addition to antipsychotics, other pharmacological treatments commonly prescribed for TS and other TDs, though not specifically investigated for sleep disturbances in TS populations, may also influence sleep. These include alpha-2 adrenergic agonists such as Clonidine, as well as central nervous system stimulants like Methylphenidate [25]. These medications are frequently used to address comorbid conditions, particularly ADHD, and may indirectly affect sleep regulation. Their impact on sleep is variable and depends on multiple factors, including the pharmacological profile of the drug, the individual’s psychiatric condition, and the presence of additional comorbidities. Consequently, treatment decisions should be individualized, with careful consideration of both tic control and sleep quality.

Nonpharmacological approaches represent an essential component of treatment and are generally recommended as first-line interventions for most sleep disorders. Cognitive behavioral therapy for insomnia (CBT-I) encompasses a range of techniques, including sleep restriction, stimulus control, cognitive restructuring, and relaxation strategies, all aimed at disrupting maladaptive sleep-related behaviors and cognitions [50,51]. CBT-I has demonstrated efficacy in improving insomnia symptoms and enhancing sleep stability by promoting a more consolidated sleep architecture, which may reduce nocturnal motor activity and parasomnias [52,53]. Although CBT-I has not yet been specifically studied in patients with TS, it may offer synergistic benefits for those experiencing concurrent sleep disturbances. Cognitive behavioral therapy more broadly has shown effectiveness in managing TS symptoms [54,55]. The shared emphasis on cognitive and behavioral modification suggests that CBT-I could simultaneously address sleep dysfunction and tic severity. Moreover, its relaxation components may help mitigate stress and muscle tension, both of which are known to exacerbate tics [56,57,58]. Mindfulness-based interventions have also shown promise in improving sleep by enhancing awareness of internal states and cognitive processes [59,60]. A pilot study examining a modified mindfulness-based stress reduction program in individuals with TS and other TDs reported that 58.8% of participants experienced significant reductions in tic severity, which were associated with increased self-awareness of factors influencing tic expression [61]. Combined with these data, our findings underscore the importance of developing individualized treatment approaches that specifically address sleep disturbances in patients with TS and other TDs.

### 4.3. Suggestions for Future Research

Future studies should focus on multimodal, longitudinal studies that combine comprehensive psychiatric and neurocognitive profiles with objective and subjective sleep evaluations. Some of the drawbacks of laboratory research could be addressed by actigraphy and home-based PSG, which could improve ecological validity while maintaining measurement accuracy. The investigation of developmental trajectories and the determination of whether specific sleep disorders remit, persist, or evolve together with tic symptoms would be made possible by large-scale cohort studies with repeated longitudinal assessments. Given the established impact of central nervous system stimulants and antidopaminergic pharmacotherapy on sleep measurements, stratified recruitment should take medication status into consideration. The ecological validity and cross-cultural generalizability of the findings could be enhanced by examining cultural and environmental factors such as bedtime practices, school start times, and co-sleeping norms. Additionally, interventional research is acutely needed to determine whether behavioral and/or pharmacological approaches to treating specific sleep disorders can result in quantifiable changes in tic severity, emotional wellbeing, and health-related quality of life. Moving beyond correlation to demonstrate causation will require adequately powered randomized controlled trials with sample sizes based on effect sizes from preliminary observational data.

## 5. Conclusions

This systematic literature review investigated the prevalence of sleep disorders in individuals with TS and other TDs, with the aim of characterizing their forms and exploring their relationships with tic severity and psychiatric comorbidities. Higher-level evidence was limited to ten studies published since 2000. Given the substantial heterogeneity among studies with respect to age, diagnostic criteria, pharmacotherapy exposure, comorbidity profiles, and sleep assessment methods, the conclusions of this review should be considered hypothesis-generating and interpreted in light of the observational study designs. Converging evidence suggests that sleep disturbances affect a significant proportion of people with TS, with prevalence estimates ranging from 32% in large-scale epidemiological data to more than 60% in clinical samples. Sleep disturbance was found to be consistently elevated relative to controls, with frequent features including sleep-onset delay, night waking/maintenance problems, and parasomnias. Discrepancies between subjective (e.g., CSHQ) and objective (e.g., PSG) measures were common, underscoring the value of multimodal assessments. Subgroup analysis demonstrates that risk is not uniform. Comorbid tic-related OCD, ADHD, anxiety and depression significantly increase insomnia, parasomnias, and night waking. With regard to tic severity, our findings, supported by PSG research, show that higher tic scores are linked with greater fragmentation, longer latency, and worse sleep efficiency, regardless of age or gender. Tics contribute to, but do not entirely cause, nocturnal impairment. Crucially, the high prevalence of sleep disorders in patients with TS and other TDs cannot be explained solely by comorbidities or pharmacological effects; intrinsic mechanisms such as cortico-striato-thalamo-cortical dysfunction and neurotransmitter imbalances are likely to influence both motor control and sleep–wake regulation. Clinically, our findings support the systematic screening of sleep disorders as part of routine diagnostic protocols for TS. Given the significant link between poor sleep, increased tic severity, and lower health-related quality of life, early detection and treatment of sleep issues should be considered as a priority in this patient population.

## Figures and Tables

**Table 1:** Cochrane Risk of Bias Assessment of the reviewed studies.

Study	Randomization	Allocation Concealment	Blinding of Participants and Personnel	Blinding of Outcome	Attrition (Incomplete Outcome Data)	Selective Reporting	Other Bias	Overall Risk
Cohrs et al. (2001) [36]	Unclear risk	Unclear risk	High risk	Unclear risk	Low risk	Low risk	High risk	High risk
Kostanecka-Endress et al. (2003) [37]	Unclear risk	Unclear risk	High risk	Unclear risk	Low risk	Low risk	Unclear risk	Unclear risk
Kirov et al. (2007) [38]	Unclear risk	Low risk	High risk	Unclear risk	Low risk	Low risk	Unclear risk	Unclear risk
Ghosh et al. (2014) [39]	Low risk	Low risk	High risk	Unclear risk	Low risk	Low risk	Unclear risk	Unclear risk
Modafferi et al. (2016) [40]	Low risk	Low risk	High risk	Unclear risk	Low risk	Low risk	Unclear risk	Unclear risk
Sambrani et al. (2016) [41]	Low risk	Low risk	High risk	Unclear risk	Low risk	Low risk	Unclear risk	Unclear risk
Ricketts et al. (2018) [42]	Low risk	Low risk	High risk	High risk	Unclear risk	Low risk	Unclear risk	Unclear risk
Isomura et al. (2022) [43]	Low risk	Low risk	High risk	Low risk	Low risk	Low risk	Low risk	Low risk
Mi et al. (2022) [44]	Unclear risk	Low risk	High risk	Unclear risk	Low risk	Low risk	Unclear risk	Unclear risk
Chung et al. (2025) [45]	Low risk	Low risk	High risk	Unclear risk	Low risk	Low risk	Low risk	Low risk

**Table 2:** Characteristics of the reviewed studies on sleep problems in patients with Tourette syndrome and other tic disorders published since 2000: polysomnography and actigraphy studies.

Study	Country	Sample	Assessment Tool(s) (Duration of Recording)	Tic Severity Measure	Pharmacotherapy	Primary Outcome	Effect Size (95% CI)	Key Findings
Cohrs et al. (2001) [36]	Germany	*n* = 25 TS (mean age 29 y; range 16–43 y; 64% M) + *n* = 14 controls	PSG (2 nights)	TSSS	12 patients	Sleep efficiency (%) in patients with TS vs. controls	Cohen’s d 1.11 (lower in TS)	Patients with TS had shorter REM latency, lower REM density, greater REM and NREM movement index, reduced sleep efficiency, longer sleep latency, higher NREM stage 1%, lower SWS %, more awakenings and sleep stage transitions. Daytime tic severity was associated with lower sleep efficiency, more awakenings, and more stage shifts.
Kostanecka-Endress et al. (2003) [37]	Germany	*n* = 17 TS (mean age 12 y; range 8–15 y; 71% M) + *n* = 16 controls	Actigraphy; PSG (2 nights)	TSSS	10 patients (discontinued before recording)	WASO (minutes) in patients with TS vs. controls	Mean difference 11.1 min (95% CI 2.4–19.8)	Patients with TS had longer sleep latency, lower sleep efficiency, and higher WASO, with more SWS arousals per hour. There was evidence of fluctuation between weekdays and weekends, as well as disruption to circadian rhythms.
Kirov et al. (2007) [38]	Germany	*n* = 54 (mean age 11 y; range 8–16 y; 92% M): 18 TD-only; 18 TD + ADHD; 18 ADHD-only + *n* = 18 controls	PSG (2 nights)	TSSS	37 patients (discontinued before recording)	REM % in each clinical subgroup vs. controls	Cohen’s d 0.45 (TD-only) vs. 0.98 (TD + ADHD) vs. 0.65 (ADHD-only)	Patients with TD had reduced sleep efficiency and increased micro-arousals in REM. Patients with ADHD had shorter sleep latency and increased REM %. Patients with TD + ADHD had additive patterns, with increased periodic limb movements and nocturnal arousals, resulting in non-restorative sleep.

Abbreviations. ADHD, attention-deficit/hyperactivity disorder; CI, confidence interval; PSG, polysomnography; SWS, slow wave sleep; TD, tic disorder; TS, Tourette syndrome; TSSS, Tourette Syndrome Severity Scale; WASO, wake after sleep onset.

**Table 3:** Characteristics of the reviewed studies on sleep problems in patients with Tourette syndrome and other tic disorders published since 2000: cross-sectional observational studies reporting subjective measures only.

Study	Country	Sample	Assessment Tool(s)	Tic Severity Measure	Pharmacotherapy	Primary Outcome	Effect Size (95% CI)	Key Findings
Ghosh et al. (2014) [39]	United States	*n* = 123 (mean age 13 y; range 6–21 y; 78% M): 48 TS-only; 75 TS + ADHD	Ad hoc sleep questionnaire	Not reported	Not reported	Sleep disorders in patients with TS-only vs. TS + ADHD	65% (TS-only) vs. 64% (TS + ADHD)	Up to 65% of patients with TS reported clinically significant sleep problems (most commonly bedtime resistance, nocturnal awakenings, and restless sleep), with sleep profiles indicative of a DSM-5 coded sleep disorder. Comorbid ADHD was associated with higher prevalence of insomnia (77% vs. 48%), problems in sleep initiation (56% vs. 48%) and sleep maintenance (47% vs. 27%). Only patients with TS + ADHD had medication-induced sleeplessness (33%).
Modafferi et al. (2016) [40]	Italy	*n* = 36 TD (mean age 12 y; age range 8–16 y; 83% M) + *n* = 266 controls	SDQ	YGTSS	11 patients	Parent-rated sleeping problems in TD vs. controls	Significant group differences across 16/45 sleep problems (effect size not reported)	Patients with TD had poorer sleep quality and higher prevalence of chronic sleep problems, especially when associated with internalizing disorders (OCD, anxiety): higher rates of insomnia and more difficulty falling asleep, greater motor activity while sleeping, higher rates of night awakenings and parasomnias (such as snoring, nightmares, and bruxism), higher levels of daytime somnolence and “falling asleep at school”.
Sambrani et al. (2016) [41]	Germany	*n* = 1032 TD (median age 17 y; range 4–72 y; 77% M), of whom *n* = 449 with comorbid ADHD and *n* = 97 with comorbid OCD	Semi-structured clinical interview	STSS	Not reported	Sleeping problems in TD-only vs. TD with any comorbidity	OR 7.08 (95% CI 2.56–19.58)	More than 1 in 4 patients with TD had a lifetime history of sleep problems (overall 26.7%: TD-only 5.3%; TD with comorbidity 28.7%). Poor sleep showed correlations with OCD, anxiety, ADHD, and depression, and was linked to lower academic achievement and higher household stress.
Ricketts et al. (2018) [42]	United States	*n* = 420 TS (age range 6–17 y; 80% M) + *n* = 254 controls	Sleep interview (telephone survey)	Parent rating (mild/moderate/severe)	257 patients	Nights/week of “sufficient sleep” in patients with TS vs. controls	Significantly fewer nights of “sufficient sleep” in TS (CI not reported)	Patients with TS had on average 1.5 fewer nights of “sufficient sleep” per week than controls. Up to 40% of patients reported insomnia, and their sleep problems (insomnia, parasomnias, hypersomnolence) were more closely associated with comorbidities than tic severity.
Mi et al. (2022) [44]	China	*n* = 271 TD (mean age 8 y; range 6–11 y; 85% M), of whom *n* = 99 with comorbid ADHD + *n* = 271 controls	CSHQ	YGTSS	Not reported	Global sleep disturbance (CSHQ total score > 41) in patients with TD vs. controls	aOR 1.95 (1.20–3.06)	Patients with TD were more likely to experience insomnia and difficulty falling asleep and had higher rates of parasomnias (24.0% vs. 7.7%), night waking (10.3% vs. 1.1%), bedtime resistance (52.8% vs. 19.2%). In some cases, sleep problems occurred before the development of tics, and were not usually linked to tic severity.

Abbreviations. ADHD, attention-deficit/hyperactivity disorder; aOR, adjusted odd ratio; CI, confidence interval; CSHQ, Children’s Sleep Habits Questionnaire; DSM-5, Diagnostic and Statistical Manual of Mental Disorders, Fifth Edition; OCD, obsessive–compulsive disorder; SDQ, Sleep Disturbance Questionnaire; STSS, Shapiro Tourette Syndrome Severity Scale; TD, tic disorder; TS, Tourette syndrome; YGTSS, Yale Global Tic Severity Scale.

**Table 4:** Characteristics of the reviewed studies on sleep problems in patients with Tourette syndrome and other tic disorders published since 2000: registry-based studies.

Study	Country	Sample	Assessment Tool(s)	Tic Severity Measure	Pharmacotherapy	Primary Outcome	Effect Size (95% CI)	Key Findings
Isomura et al. (2022) [43]	Sweden	*n* = 5877 TD (age ≥ 3 y; 78% M), of whom *n* = 3130 with comorbid ADHD and *n* = 1106 with comorbid OCD + *n* = 10,438,825 controls	Clinical observation + use of insomnia medications	Not reported	2568 patients on ADHD medications	Diagnosis of insomnia in patients with TD vs. controls	aOR 6.74 (6.37–7.15)	Patients with TD had higher rates of insomnia than controls (32.2% vs. 13.7%). Bedtime reluctance and sleep anxiety were among the most commonly reported issues alongside insomnia. Comorbid ADHD and OCD, but not tic severity, predicted sleep problems.
Chung et al. (2025) [45]	Taiwan	*n* = 13,646 TS (mean age 11 y; 83% M), of whom *n* = 2260 with comorbid ADHD and *n* = 188 with comorbid OCD + *n* = 54,584 controls	Clinical observation	Not reported	Not reported	Sleep disorders in patients with TS vs. controls	aHR 1.76 (1.58–1.96)	The incidence of sleep disorders was highest within 1 year of TS diagnosis (aHR 3.68) and decreased with age. RBD-like symptoms were found in three out of ten patients with TS and included complex motor patterns (e.g., arm flailing and talking). Increased risk of sleep disorders in patients with comorbid ADHD and anxiety.

Abbreviations. ADHD, attention-deficit/hyperactivity disorder; aHR, adjusted hazard ratio; aOR, adjusted odd ratio; OCD, obsessive–compulsive disorder; RBD, REM sleep behavior disorder; TD, tic disorder; TS, Tourette syndrome.

**Table 5:** Sleep problems most commonly reported in patients with Tourette syndrome and other tic disorders.

Sleep Problem	Manifestations in TS/TD	Proposed Pathophysiology	Key Studies
Insomnia	Difficulty initiating/maintaining sleep; frequent night-time awakenings	Heightened arousal, dopaminergic dysregulation, and concomitant anxiety/OCD	Sambrani et al. (2016) [41]; Isomura et al. (2022) [43]; Ricketts et al. (2018) [42]
Sleep onset delay	Prolonged time to fall asleep	Pre-sleep tics, restlessness, rumination	Mi et al. (2022) [44]
Night awakenings	Multiple episodes of awakening during the night	Tic expression during lighter sleep stages, reduced sleep efficiency	Cohrs et al. (2001) [36]; Ghosh et al. (2014) [39]
Restless sleep	Tossing, turning, moving limbs; sleep instability	Sleep-related movements, possible comorbidity with restless legs syndrome	Kirov et al. (2007) [38]
Daytime sleepiness/hypersomnolence	Fatigue, inattention, difficulty waking	Disrupted sleep architecture, reduced REM, insufficient total sleep time	Modafferi et al. (2016) [40]
Parasomnias	Somnambulism, night terrors, vocalizations	Overlap with motor/vocal tics; confusion with nocturnal seizures or REM behavior disorder	Ghosh et al. (2014) [39]; Mi et al. (2022) [44]
REM behavior disorder	Acting out dreams, jerky movements during REM sleep	REM atonia breakdown possibly linked to dopaminergic medications	Chung et al. (2025) [45]
Circadian rhythm disorders	Irregular sleep timing, delayed sleep phase	Behavioral dysregulation, poor sleep hygiene, comorbid ADHD	Ricketts et al. (2018) [42]
Sleep-related tic exacerbation	Persistence or exacerbation of tics during lighter NREM sleep stages	Tics may persist into sleep; differentiate from nocturnal seizures	Cohrs et al. (2001) [36]; Kirov et al. (2007) [38]
Sleep anxiety/bedtime resistance	Anxiety at bedtime, resistance to sleep routines	Anticipation of sleep disruptions, underlying anxiety	Sambrani et al. (2016) [41]
Reduced REM/altered sleep architecture	Shortened REM duration, increased wake after sleep onset	Neurophysiological immaturity, possible medication effects	Cohrs et al. (2001) [36]; Kostanecka-Endress et al. (2003) [37]

Abbreviations. ADHD, attention-deficit/hyperactivity disorder; OCD, obsessive–compulsive disorder; TD, tic disorder; TS, Tourette syndrome.

## Data Availability

The original contributions presented in this study are included in the article. Further inquiries can be directed to the corresponding author.

## Supplementary Materials

The following supporting information can be downloaded at: https://www.mdpi.com/article/10.3390/jpm16060309/s1, File S1: PRISMA 2020 Checklist.
